# Evaluation of genetic parameters for growth and cold tolerance traits in *Fenneropenaeus chinensis* juveniles

**DOI:** 10.1371/journal.pone.0183801

**Published:** 2017-08-29

**Authors:** Mingzhu Wang, Jie Kong, Xianhong Meng, Sheng Luan, Kun Luo, Juan Sui, Baolong Chen, Jiawang Cao, Xiaoli Shi

**Affiliations:** 1 Key Laboratory of Sustainable Utilization of Marine Fisheries Resources, Ministry of Agriculture, Yellow Sea Fisheries Research Institute, Chinese Academy of Fishery Sciences, Qingdao, China; 2 Laboratory for Marine Fisheries Science and Food Production Processes, Qingdao National Laboratory for Marine Science and Technology, Qingdao, China; 3 Shanghai Ocean University, Shanghai, China; China Agricultural University, CHINA

## Abstract

In this study, genetic parameters were obtained for growth and cold tolerance of 99 *Fenneropenaeus chinensis* juvenile families by means of indoor artificial cooling (starting from 14°C, 2°C/d). A linear mixed model was fitted to estimate variance components using the ASReml software package. Heritabilities estimated for body weight (BW) and body length (BL) of *F*. *chinensis* juveniles were 0.078 ± 0.124 and 0.131 ± 0.133, respectively. The estimates of heritability were low in magnitude for both traits. The differences between the heritabilities estimated for the two growth traits were not significant with each other, and the heritabilities were not significantly different from zero (*P* > 0.05). The phenotypic and genetic correlation coefficients between BW and BL were as high as 0.9408 ± 0.0040 and 0.9562 ± 0.0551, respectively, and both were significantly different from zero (*P* < 0.01). The heritabilities of temperature at death (TAD) and cooling degree hours (CDH) were 0.265 ± 0.091 and 0.077 ± 0.058, respectively. The heritability estimates for TAD was moderate and significantly different from zero (*P* < 0.01). The correlation analysis indicated that the phenotypic correlation coefficient between TAD and CDH was -0.5470 ± 0.0174, and the genetic correlation coefficient was -0.6707 ± 0.3635. In the analysis of growth traits and cold tolerance traits, the values of phenotypic correlation coefficient were floating between -0.1055 and 0.1098, both were significantly different from zero (*P* < 0.05), while the genetic correlation had a larger range (0.0526 ~ 0.9914), and all were not significantly different from zero (*P* > 0.05). In this study, there was a low correlation between growth and cold tolerance traits, indicating that growth traits and cold tolerance traits should be considered collectively in the breeding program of shrimp.

## Introduction

*Fenneropenaeus chinensis* is one of the most representative indigenous aquaculture species of China, and it is mainly distributed in the Yellow Sea and Bohai Sea area, and was cultured in Shandong, Hebei, Liaoning, Tianjin, Jiangsu offshore, as well as west coast and south coast of the Korean Peninsula. The optimum temperature ranges from 18 to 30°C for *F*. *chinensis* and the most suitable temperature is 25°C. The tolerance range of water temperature ranges from 4 to 38°C [1]. From the 1970s, factory seedling of *F*. *chinensis* was gradually dealt with. In the 1980s, the breeding area continued to expand. During the period from 1988 to 1993, the production of *F*. *chinensis* consecutively ranked the first in the world and the annual yield was as high as more than 200,000 t [1, 2], which brought huge economic benefit to aquaculture industry. However, in 1993 the *F*. *chinensis* farming industry suffered from white spot syndrome virus (WSSV), which resulted in serious economic losses to shrimp farming industry. In 1994 national production of shrimp sharply fell to around 60,000 t [2]. Facing the difficulty in the farming of shrimp, breeding staff, after a long struggle, developed 3 new varieties of Chinese shrimp namely “Huanghai No. 1” (2003), “Huanghai No. 2” (2008) and “Huanghai No. 3” (2013) [3–5], which enabled gradual recovery of China’s farming of *F*. *chinensis*. However, in recent years, new problems have arisen in shrimp farming. The intensified abnormal climate especially repeated cold disaster nationwide has resulted in mortality of a large number of shrimps and serious economic losses to shrimp farmers; on the other hand, the optimum temperature of *F*. *chinensis* restrains breeding season and region, and further restrains pond stocking time and harvest specifications of shrimp, and indirectly affects yield and benefit of breeding, and therefore, the breeding of cold tolerance varieties is expected to solve all the above problems.

The study on evaluation of genetic parameters of temperature tolerance of aquatic animals has been carried out for many years. Charo-Karisa et al. [6] studied on cooling degree hours (CDH) of Nile Tilapia (*Oreochromis niloticus*) and temperature at death (TAD) at low temperature. Liu Baosuo et al. [7] and Perry et al. [8] studied the upper thermal tolerance of *Scophthalmus maximus* and rainbow trout (*Oncorhynchus mykiss*), respectively. In addition, Cnaani et al. [9] also studied the cold tolerance of tilapia hybrid variety. There have been not too many studies of genetic parameters of temperature tolerance up to now. There is only one study of Li Wenjia et al. [10, 11] on the genetic parameters of adult Chinese shrimp and *Litopenaeus Vannamei* on the genetic parameters of cold tolerance. However, there is no report on cold tolerance traits in *F*. *chinensis* Juveniles.

In this study, a preliminary evaluation of growth and cold tolerance traits of 99 *F*. *chinensis* juvenile families was made by means of indoor artificial cold tolerance challenge with analysis of heritability of body weight (BW), body length (BL), CDH and TAD. It was expected to provide a theoretical basis for breeding of cold tolerance variety of *F*. *chinensis*. The lowest temperature for survival of *F*. *chinensis* is 4°C and breeding temperature is usually not lower than 14°C [1], and therefore water temperature in this experiment ranges from 4 to 14°C.

## Materials and methods

### Ethical statement

This study was approved by the Animal Care and Use committee in the Yellow Sea Fisheries Research Institute, Chinese Academy of Fishery Sciences. The selection of the shrimps in the experiments of this study was in accordance with the Law of the People’s Republic of China on the Protection of Wildlife (http://www.china.org.cn/english/environment/34349.htm).

### Experimental materials

The experiment was conducted in the Mariculture Research Station of Yellow Sea Fisheries Research Institute, Chinese Academy of Fishery Sciences (N36°20’32.51”, E120°38’59.69” and altitude 3.04 m), located in Qingdao City, Shandong Province, China. The experimental materials were 99 “Huanghai No. 2” families of *F*. *chinensis* built in 2015 (including 12 half-sib families), representing the G_10_-generation, at age of 40 days. 30 individuals were randomly sampled from each family at the average body weight of 0.073 ± 0.043g and body length of 19.790 ± 3.607mm, respectively.

### Cold tolerance challenge treatment of juveniles

30 juvenile shrimps were randomly sampled from each family and placed in a storage box sized 26.5 cm×20 cm×16.5 cm. An air stone was put in each box. A group of 3 boxes was placed in a refrigerator box sized 72 cm×37 cm×20 cm. Finally, the refrigerator box was placed in a refrigerator, sized 183 cm×84 cm×92 cm, with adjustable temperature. As the breeding temperature in nursery workshop was 24°C, temperature was dropped to 14°C at a rate of 2°C/d for juveniles. Then experiment was started. During the experiment, the temperature was dropped to 4°C at a rate of 2°C/d and maintained at 4°C till all the juvenile shrimps were dead. During the experiment, the water temperature was monitored per hour to ensure the temperature fluctuated less than 1°C. The juvenile shrimps were fed 4 times. The daily water exchange was 30%. From the first mortality of shrimp, the storage box was checked every 2 hours. Dead shrimps were collected and information like mortality time, family, temperature at mortality was recorded, body weight and body length were measured for each shrimp. The CDH was calculated according to the experimental results and the initial temperature for the calculation of CDH was 14°C. The accuracy of analytical balance was 0.001g and the accuracy of vernier caliper was 0.01mm.

### Data processing

The experimental data were recorded in Excel, and descriptive statistics were performed using the SPSS19.0 (SPSS Inc., Michigan Avenue, Chicago, IL, USA) for Windows.

#### Estimates of heritability and correlation analysis of growth traits in *F*. *chinensis* juveniles

In this study, a liner mixed model was fitted with average information restricted maximum likelihood (AIREML). ASReml Software was applied to estimate the variance components of body weight and body length of *F*. *chinensis*. For body weight, the normal test of residuals showed that the data were not normally distributed and transformed by natural logarithms (Ln). It proved that all the fixed effects and covariates were statistically significant (*P* < 0.01). Variance components were estimated with a univariate mixed liner animal model. The animal model was written as follows:

$$Y_{ij}=\mu +b*{Age}_{i}+a_{i}+f_{j}+e_{ij}$$ (1)

Where *Y*_*ij*_ is the observed value of BW or BL of the *i*^th^ shrimp, *μ* is the overall mean. Age_*i*_ is the covariate of the *i*^th^ individual age at the end of the experiment, *b* is the regression coefficient, *a*_*i*_ is the additive genetic effect of BW or BL of the *i*^th^ shrimp, *f*_*j*_ is the common environment effects of separate breeding of full-sib families and *e*_*ij*_ is the random residual error of the *i*^th^ shrimp.

Phenotypic variance is the sum of all variance components, and calculation formula was as follows:

$$\sigma_{p}^{2}=\sigma_{a}^{2}+\sigma_{e}^{2}+\sigma_{f}^{2}$$ (2)

Heritability is the ratio of additive genetic variance to phenotypic variance, and calculation formula was as follows:

$$h^{2}=\sigma_{a}^{2}/\sigma_{p}^{2}$$ (3)

Where $\sigma_{p}^{2}$ is phenotypic variance, $\sigma_{a}^{2}$ is additive variance, $\sigma_{e}^{2}$ is residual fraction, and $\sigma_{f}^{2}$ is common environment variance.

A bivariate animal model was fitted to estimate the phenotypic and genetic correlations between body weight and body length was using the ASReml software package. The description for each term of the models was the same as that for Model (1).

#### Estimates of heritability and correlation analysis of cold tolerance traits in *F*. *chinensis* juveniles

In this study, TAD and CDH are cold tolerance parameters of *F*. *chinensis*. TAD refers to temperature at death. CDH represents the sum of hours the shrimp has survived multiplied by the difference between final and initial temperature for each shrimp. The initial temperature for calculation of CDH was set at 14°C. The formula was as follows [9, 12]:

$$CDH=\sum_{i=1}^{k}\left[t_{i}\times (T_{0}-T_{i})\right]$$ (4)

Where: *t*_*i*_ *=* the cumulated hours before temperature drop, *T*_0_ = 14°C, the initial temperature, *T*_*i*_ = temperature at the *i*^*th*^ hour, and *k* = the cumulated hours at mortality.

The variance components of CDH and TAD were estimated with a univariate mixed liner animal model analyses using ASReml Program. Age has a linear correlation with CDH and TAD (*P* < 0.01), which are used as covariates accordingly. All the fixed effects and covariates were statistically significant (*P* < 0.01). The model was as follows:

$$Y_{ijk}=\mu +b*{Age}_{i}+a_{i}+{Tank}_{j}+f_{k}+e_{ijk}$$ (5)

Where *Y*_*ijk*_ is the observed value of CDH or TAD of the *i*^th^ shrimp, *μ* is the overall mean, Age_*i*_ is the covariate of the *i*^th^ individual age at the end of the experiment, *b* is the regression coefficient, *a*_*i*_ is the additive genetic effects of CDH or TAD of the *i*^th^ shrimp, *Tank*_*j*_ is the fixed effect of the *j*^th^ refrigerator, *f*_*k*_ is the common environment effect of separate breeding of the *k*^th^ full-sib family, and *e*_*ijk*_ is the random residual error of the *i*^th^ shrimp.

In model (5), the estimated values of heritability of CDH and TAD were affected by temperature. Temperature varied among different refrigerators, so refrigerator effect was regarded to be fixed effect. Calculation formulas of phenotypic variance and heritability essentially agreed with those described in formula (2) and formula (3).

Similarly, the phenotypic and genetic correlations between CDH and TAD were estimated in bivariate animal model analyses using ASReml package. The description for each term of the models was the same as that for Model (5).

#### Correlation analysis of growth traits and cold tolerance traits in *F*. *chinensis* juveniles

ASReml Program is applied in the phenotypic and genetic correlation analysis of growth and cold tolerance in *F*. *chinensis*, and the specific methods are described in Model (1) and Model (5). Bivariate animal model is applied in the correlation analysis of BW / TAD, BW / CDH, BL / TAD and BL / CDH.

#### Significance test of heritability and correlation evaluation

Z-score is applied to test the significance of the above heritability and correlation evaluation and the formula was as follows [13]:

$$Z=\frac{x_{i}-x_{j}}{\sqrt{\sigma_{i}^{2}+\sigma_{j}^{2}}}$$ (6)

Where *x*_*i*_ and *x*_*j*_ are heritability correlation and genetic correlation, respectively, and $\sigma_{i}^{2}$ and $\sigma_{j}^{2}$ are standard errors of heritability correlation and genetic correlation accordingly. Both of *x*_*j*_ and *σ*_*j*_ are defined as zero when genetic parameter is tested for its difference from zero.

## Results

### Descriptive statistics of growth and cold tolerance of *F*. *chinensis* juveniles

**Fig 1:**
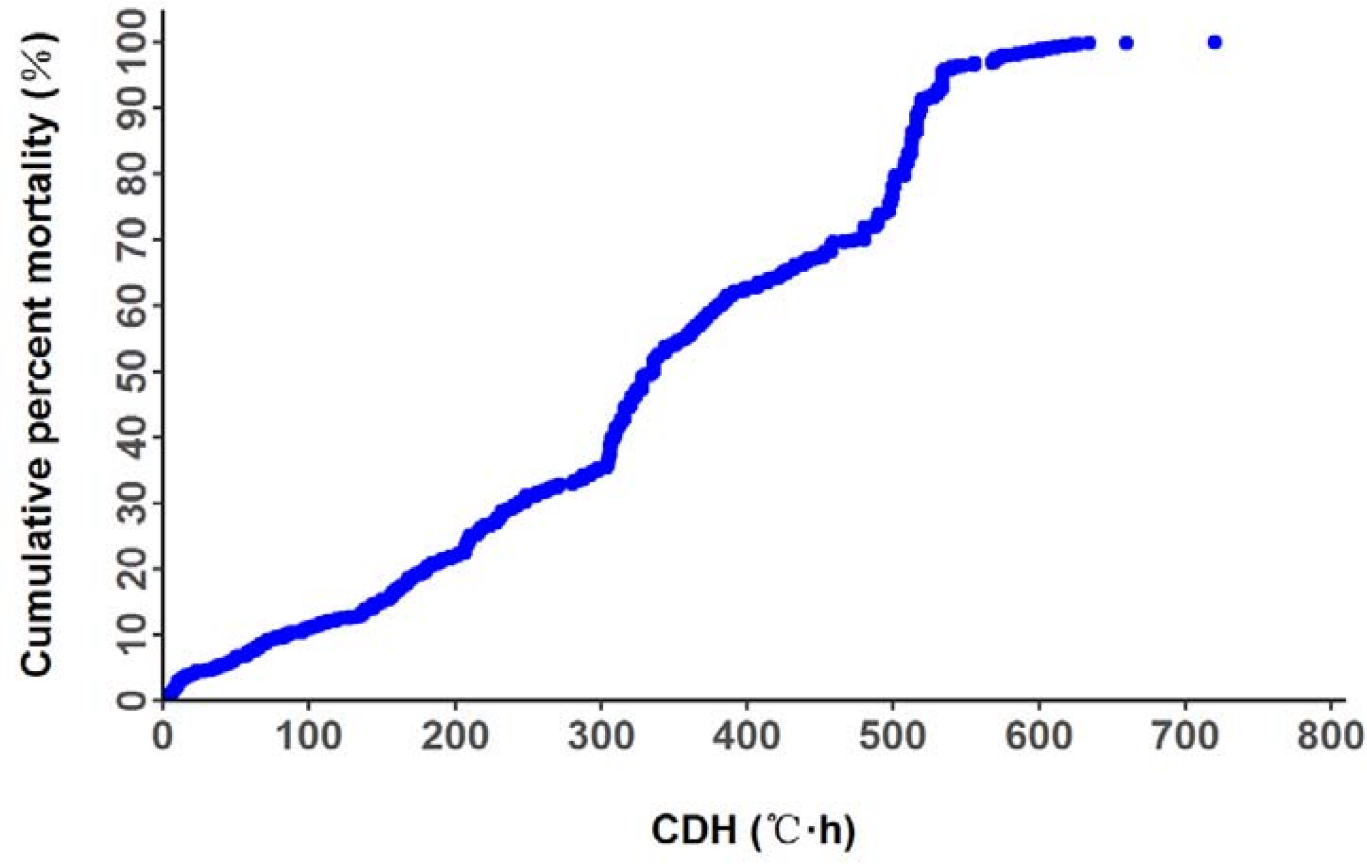
Cumulative mortality of *F*. *chinensis* juveniles at different CDH values during the cold tolerance challenge.

The sample size, mean, maximum, minimum, standard deviation and coefficient variation for growth and cold tolerance traits of *F*. *chinensis* are shown in Table 1. The mean of BW, BL, TAD, CDH and survival rate for each family at half lethal time (SR_50_) were 0.073g, 19.790mm, 6.506°C, 333.937°C•h, 48.950%, respectively, with coefficient variation ranging from 18.226% to 59.919%. The coefficient variation of body weight was the greatest (59.919%), indicating that there was great variance in body weight between different families. while the coefficient variation of body length was the smallest (18.226%), indicating that there was comparatively small variance in body length between different families. Among all the cold tolerance traits, SR_50_ had the highest phenotypic variance, with 54.107% coefficient variation. It was followed by CDH (48.221%), with TAD as the lowest (36.241%). In cold tolerance challenge experiment, mortality of shrimp was observed when water temperature was 13.9°C and CDH was 0.800°C•h. All the shrimps were dead when CDH reached 720.000°C•h (Fig 1). The cumulative mortality curve was shown in Fig 1.

**Table 1:** Description statistics of growth and cold tolerance traits in *F*. *chinensis* juveniles.

Traits	Number	Mean	Minimum	Maximum	Standard deviation	Coefficient variation (%)
**BW (g)**	2508	0.073	0.005	0.370	0.044	59.919
**BL (mm)**	2508	19.790	9.610	33.120	3.607	18.226
**CDH (°C·h)**	2508	333.937	0.800	720.000	161.030	48.221
**TAD (°C)**	2508	6.506	3.000	14.000	2.358	36.241
**SR**_**50**_ **(%)**	99	48.950	0	100.000	26.485	54.107

### Estimates of genetic parameters for growth traits in *F*. *chinensis* juveniles

The variance components and heritability for growth traits of *F*. *chinensis* juveniles are presented in Table 2. The heritability estimates for BW and BL were 0.078 ± 0.124 and 0.131 ± 0.133, respectively, and both were not significantly different from zero (*P* > 0.05). According to Z-score test, there was no significant difference between BW and BL in terms of heritability (*Z* = 0.2904, *P* > 0.05). As shown in Table 3, the phenotypic and genetic correlation between BW and BL were as high as 0.9408 ± 0.0040 and 0.9562 ± 0.0551, respectively, which were significantly different from zero (*P < 0*.*01*). These results indicated that growth traits (BW and BL) were highly correlated.

**Table 2:** Variance components, heritability of growth and cold tolerance traits in *F*. *chinensis* juveniles.

Traits		Variance components			Heritability
	$\sigma_{a}^{2}$	$\sigma_{f}^{2}$	$\sigma_{e}^{2}$	$\sigma_{p}^{2}$	*h*^2^ ± SE
**LnBW**	0.026	0.116	0.194	0.337	0.078 ± 0.124
**BL**	1.590	4.150	6.420	12.160	0.131 ± 0.133
**TAD**	0.368	0.001	1.021	1.390	0.265 ± 0.091**
**CDH**	266.000	151.000	3043.000	3460.000	0.077 ± 0.058

$\sigma_{a}^{2}$ is the additive genetic variance, $\sigma_{f}^{2}$ is the common environment variance, $\sigma_{e}^{2}$ is the random residual error variance, $\sigma_{p}^{2}$ is the phenotypic variance, *h*^2^ is the heritability. SE is the standard errors of heritability.

** Estimate was significantly different from zero (*P* < 0.01).

**Table 3:** Phenotypic and genetic correlations for growth and cold tolerance traits of *F*. *chinensis* juveniles.

Traits	BW	BL	TAD	CDH
**BW**	1	0.9562 ± 0.0551**	0.1391 ± 0.6297	0.5856 ± 0.8421
**BL**	0.9408 ± 0.0040**	1	-0.0526 ± 0.4664	0.9914 ± 0.7010
**TAD**	-0.0760 ± 0.0317*	-0.1055 ± 0.0325**	1	-0.6707 ± 0.3635
**CDH**	0.0953 ± 0.0285**	0.109 ± 0.0291**	-0.5470 ± 0.0174**	1

The parts above diagonal mean genetic correlations (and their standard errors); the parts below diagonal mean phenotypic correlation (and their standard errors).

** Estimate was significantly different from zero (*P* < 0.01)

* Estimate was significantly different from zero (*P* < 0.05).

### Evaluation of genetic parameters for cold tolerance traits in *F*. *chinensis* juveniles

The genetic parameters of cold tolerance, namely TAD and CDH, are shown in Table 2. The estimates of heritability for TAD and CDH were 0.265 ± 0.091 and 0.077 ± 0.058, respectively. The heritability of TAD was significantly different from zero (*P* < 0.01). There was no significant difference between CDH and TAD (*Z* = 1.74, *P* > 0.05). Correlation analysis indicated that phenotypic correlation between TAD and CDH was -0.5470 ± 0.0174, and genetic correlation was -0.6707 ± 0.3635 (Table 3).

### Correlation analysis of growth traits and cold tolerance traits in *F*. *chinensis* juveniles

The genetic and phenotypic correlations between growth traits (BW and BL) and cold tolerance traits (TAD and CDH) were shown in Table 3. The values of phenotypic correlation coefficients ranged from -0.1055 to 0.1098. The phenotypic correlation coefficients between growth traits (BW and BL) and cold tolerance traits (TAD and CDH) were low. The genetic correlation coefficients between growth traits and TAD were low too, whereas the genetic correlation coefficients between growth-related traits and CDH were comparatively high. The genetic correlation coefficient between BL and CDH was the highest (0.9914 ± 0.7010), while the genetic correlation coefficient between BL and TAD was the lowest (-0.0526 ± 0.4664). In this study, the phenotypic and genetic correlation coefficients between growth and cold tolerance traits of *F*. *chinensis* had a wide floating range.

## Discussion

### Heritability for growth traits of *F*. *chinensis* juveniles

The research results showed that the heritabilities for body weight and body length of *F*. *chinensis* juveniles were low (0.078 ± 0.124 and 0.131 ± 0.133, respectively). It essentially agreed with that of body weight of shrimp at the age of 150 days estimated by means of intra group correlation of full-sib by He Yuying et al. [14], whereas it was lower than heritability of body length (0.36 ~ 0.51). It essentially agreed with the result of heritability for growth traits of *F*. *chinensis* at the age of 145 days by Tian Yi et al. [15]. The heritabilities for body weight and body length of the latter were 0.14 ± 0.16 and 0.10 ± 0.06, respectively. Huang Fuyou et al. [16] evaluated the heritability for body length of *F*. *chinensis* “Huanghai No. 1” at 3 and 4 months of age by means of intra group correlation of full-sib and the estimates were 0.4 and 0.5, respectively, which showed high heritability. Zhang Tianshi et al. [17] analyzed the heritability for body weight of adult Chinese shrimp by means of 8 models, with estimates ranging from 0.44 to 0.74, which also showed high heritability. Li Wenjia et al. [10] estimated the heritability for body weight of adult Chinese shrimp under cold tolerance challenge by means of two animal models. The heritability for body weight was 0.3271 ± 0.0447 without common environment effects, and 0.1320 ± 0.0269 with common environment effects. Both were significantly different from zero (*P <* 0.01). There was significant difference between heritabilities by means of two models (*Z* = 3.7397, *P* < 0.01).

The heritabilities for growth traits obtained in the above studies were higher than those obtained in this study. The following reasons accounted for the results: 1. The heritability estimates were affected by different varieties or different growth periods (age) of the same variety, and different genetic backgrounds and structures of breeding populations [18]. The research population in above studies was adult *F*. *chinensis*, while in this study was juvenile. Also, at different growth stages, different variance components had different proportions. Common environment effect was due to separate breeding of different families, and was an important variance component in the estimate of genetic parameters. It played a more important role in the genetic parameter evaluation model in this study. 2. Different estimates of heritability were generated by environment effect and unpredictable heritability effect in analysis, due to different estimate methods [14, 18].

### Heritability for cold tolerance traits of *F*. *chinensis* juveniles

TAD and CDH were selected as parameters for cold tolerance traits in *F*. *chinensis* juveniles. The heritability estimates for TAD and CDH were 0.265 ± 0.091 and 0.077 ± 0.058, respectively, which were moderate and low in magnitude. The heritability for cold tolerance traits of *F*. *chinensis* juveniles have not been reported. Li Wenjia et al. [11] evaluated the heritability of cold tolerance traits like CDH in *L*. *vannamei* adults. It is low with the estimate of 0.0258 ± 0.0205. In other related researches of aquatic animal, Charo-Karisa et al. [6] studied the heritability for cold tolerance traits of *O*. *niloticus* juveniles and found the heritabilities for CDH and TAD was as low as 0.08 ± 0.19 and 0.09 ± 0.17, respectively. The heritability for CDH essentially agreed with the results in this study, and the heritability for TAD was lower. Liu Baosuo et al. [7] studied the heritability of hot tolerance traits in *Scophthalmus maximus* by means of two models, the heritability estimates were 0.026 ± 0.034 and 0.026 ± 0.053, respectively, which also showed low heritability. The above results also indicated that temperature tolerance traits in aquatic animals were low heritability traits. CDH considered temperature at death and time at mortality at the same temperature, which is more accurate than parameters like single temperature and time, and therefore, CDH is an accurate indicator of cold tolerance traits [7, 12].

### Correlation analysis of growth and cold tolerance traits of *F*. *chinensis* juveniles

The results in this study showed that the phenotypic correlations between growth and cold tolerance were low in *F*. *chinensis* juveniles. The genetic correlation has a larger range (0.0526 ~ 0.9914), and all were not significantly different from zero (*P* > 0.05) due to large standard errors. The results essentially agreed with the results of Li Wenjia et al. [11] and Charo-Karisa et al. [6], who studied genetic correlation between growth and cold tolerance traits of *L*. *vannamei* and genetic correlation between body weight and cold tolerance traits of Nile tilapia, respectively. The results of the Nile tilapia showed the genetic correlation between body weight and CDH was 0.72 ± 0.81, while genetic correlation between body weight and TAD was -0.68 ± 0.84, which was highly negative. In the study of hot tolerance traits in *Scophthalmus maximus*, a positive phenotypic correlation between body weight and upper thermal tolerance (UTT) was observed, but the genetic correlation was negative [7]. Due to gene linkage effect and gene pleiotropism, the degree of correlation between traits differed from each other. The traits could be selected indirectly through traits with higher correlation. In this study, there was a low correlation between growth and cold tolerance traits, indicating that growth and cold tolerance should be considered collectively in the breeding program of shrimp. In short, further studies are required for the correlation of different traits of *F*. *chinensis*, so as to provide more accurate data for breeding of cold tolerance *F*. *chinensis*. In addition, based on traditional breeding methods, traits of low heritability could be improved by means of studies of molecular biology to accelerate the breeding process [19].
